# Evaluation of Osteogenesis and Angiogenesis of Icariin in Local Controlled Release and Systemic Delivery for Calvarial Defect in Ovariectomized Rats

**DOI:** 10.1038/s41598-017-05392-z

**Published:** 2017-07-11

**Authors:** Yuqiong Wu, LingYan Cao, Lunguo Xia, Qianju Wu, Jie Wang, Xiao Wang, Lianyi Xu, Yuning Zhou, Yuanjin Xu, Xinquan Jiang

**Affiliations:** 1 Oral Bioengineering and Regenerative Medicine Lab, Shanghai Key Laboratory of Stomatology, Ninth People’s Hospital Affiliated to Shanghai Jiao Tong University, School of Medicine, 639 Zhizaoju Road, Shanghai, 200011 China; 2 Center of Craniofacial Orthodontics, Department of Oral and Maxillofacial Surgery, Ninth People’s Hospital Affiliated to Shanghai Jiao Tong University, School of Medicine, 639 Zhizaoju Road, Shanghai, 200011 China; 3 Xiamen Stomatology Hospital Affiliated to Fujian Medical University, Xiamen, 361000 Fujian Province China; 4 Department of Oral Surgery, Ninth People’s Hospital, College of Stomatology, Shanghai Jiao Tong University School of Medicine, Shanghai Key Laboratory of Stomatology and Shanghai Research Institute of Stomatology, Shanghai, China

## Abstract

Typically, bone regenerative medicine is applied to repair bone defects in patients with osteoporosis. Meanwhile, there is an urgent need to develop safe and cheap drugs that induce bone formation. Icariin, which is reported to promote the osteogenesis of stem cells *in vitro*, is the main active component of *Herba Epimedii*. However, whether icariin could repair bone defects caused by osteoporosis remains unknown. In this study, an osteoporosis model in rats was established by an ovariectomy first, and then, the osteogenic and angiogenic differentiation of bone mesenchymal stem cells (BMSCs) treated with icariin was evaluated. Furthermore, calcium phosphate cement (CPC) scaffolds loaded with icariin were constructed and then implanted into nude mice to determine the optimal construction. To evaluate its osteogenic and angiogenic ability *in vivo*, this construction was applied to calvarial defect of the ovariectomized (OVX) rats accompanied with an icariin gavage. This demonstrated that icariin could up-regulate the expression of osteogenic and angiogenic genes in BMSCs. Meanwhile, osteoclast formation was inhibited. Moreover, CPC could act as a suitable icariin delivery system for repairing bone defects by enhancing osteogenesis and angiogenesis, while the systemic administration of icariin has an antiosteoporotic effect that promotes bone defect repair.

## Introduction

Osteoporosis, a chronic disease worldwide, is characterized by the progressive loss of bone mass and microarchitectural deterioration, both of which lead to increased bone fractures and bone defect risks^1^. Drugs for the prevention and treatment of osteoporosis approved by the Food and Drug Administration (FDA) are osteogenesis promotion medicines (anabolic agents) or osteoclast depressants (anti-catabolic agents), including estrogens in the case of hormone replacement therapy (HRP) and selective estrogen receptor modulators, such as raloxifene, bisphosphonates and calcitonin^2^. However, all of these drugs are usually associated with adverse effects, such as endometrial and breast cancer, menstruation, and thromboembolic events^3^. Furthermore, there are significant challenges associated with the treatment of bone defects caused by osteoporosis. The need for new treatments of serious bone defects has promoted the emergence of tissue engineering strategies. A typical tissue engineering strategy includes cells, biomaterial scaffolds and bioactive molecules^4^. Multi-potent stem cells are widely used for bone regeneration^5^. With regard to biomaterial scaffolds, an ideal one should meet numerous requirements, including excellent biocompatibility and superior osteoconductivity and osteoinductive properties. However, most scaffolds currently used (i.e., those made from ceramics, polymers, and metals) only have osteoconductive abilities and thus lack powerful osteoinductive properties^6, 7^. Therefore, to overcome this issue, bioactive molecules, such as growth factors or cytokines, have been introduced into scaffolds^8–10^.

*Herba Epimedii* is a traditional Chinese medicine herb that has been commonly used as a tonic and aphrodisiac, as well as an anti-rheumatic and anti-osteoporotic agent in China for thousands of years^11^. It has also been demonstrated to be an effective enhancer of bone healing^12^. Icariin (C_33_H_40_O_15_; molecular weight: 676.67), a flavonoid glycoside that is the major active ingredient of *Herba Epimedii*, is considered as the standard for its quality control^13^. It corrects the decrease of estrogen in serum and partly restores the decreased weight of the uterus in ovariectomized (OVX) rats^14^. Several reports have demonstrated that icariin could enhance the osteogenic differentiation of bone mesenchymal stem cells (BMSCs)^15–17^, inhibit osteoclast differentiation, prevent ovariectomy-induced bone loss and reduction in femoral and tibia strength^14^, and promote bone formation of mandibular distraction^18^. Moreover, icariin plays a significant role in promoting the activity of human endothelial cells *in vitro*, leading to an enhanced vascularization effect^19^. However, there have not been any studies focused on the *in vivo* use of icariin for repairing bone defects caused by osteoporosis.

In the present study, we hypothesized that icariin could induce osteogenic and angiogenic factor expression in BMSCs and inhibit osteoclast formation. A calcium phosphate cement (CPC) scaffold was used to construct an icariin delivery system and was loaded with BMSCs to effectively promote bone and vessel formation in bone defects. To verify our hypothesis, the optimal concentration of icariin for osteogenic and angiogenic factor expression and osteoclast formation of BMSCs was explored by real-time polymerase chain reaction (PCR), alkaline phosphatase (ALP) activity assay and tartrate-resistant acid phosphatase (TRAP) staining. Then, CPC scaffolds loaded with icariin at different concentrations were planted in the dorsal subcutaneous pockets of nude mice to determine the optimal concentration of icariin *in vivo*. Finally, the optimized CPC/icariin construction was filled in the OVX rat calvarial defect model accompanied by an oral administration of icariin, and then, osteogenesis and angiogenesis were investigated *in vivo* by sequential fluorescent labeling, a histological assay and a micro-CT measurement 8 weeks after the implantation.

## Results

### Icariin enhanced osteogenic differentiation and angiogenic factor expression in BMSCs

In this study, BMSCs derived from OVX rats were treated in advance with icariin at a series of concentrations to confirm an appropriate concentration for *in vitro* osteogenesis by detecting cell proliferation, osteogenesis activity and angiogenic factor expression in the BMSCs. Based on our previous study^15^, icariin, at concentrations from 10 to 40 μM demonstrates an excellent ability to improve osteogenic differentiation and angiogenic factor expression in BMSCs derived from young rats, especially at 20 μM, while cell proliferation is inhibited. According to the present results, for the BMSCs derived from OVX rats, icariin concentrations of 10, 20 and 40 μM, were selected for detection.

**Figure 1:**
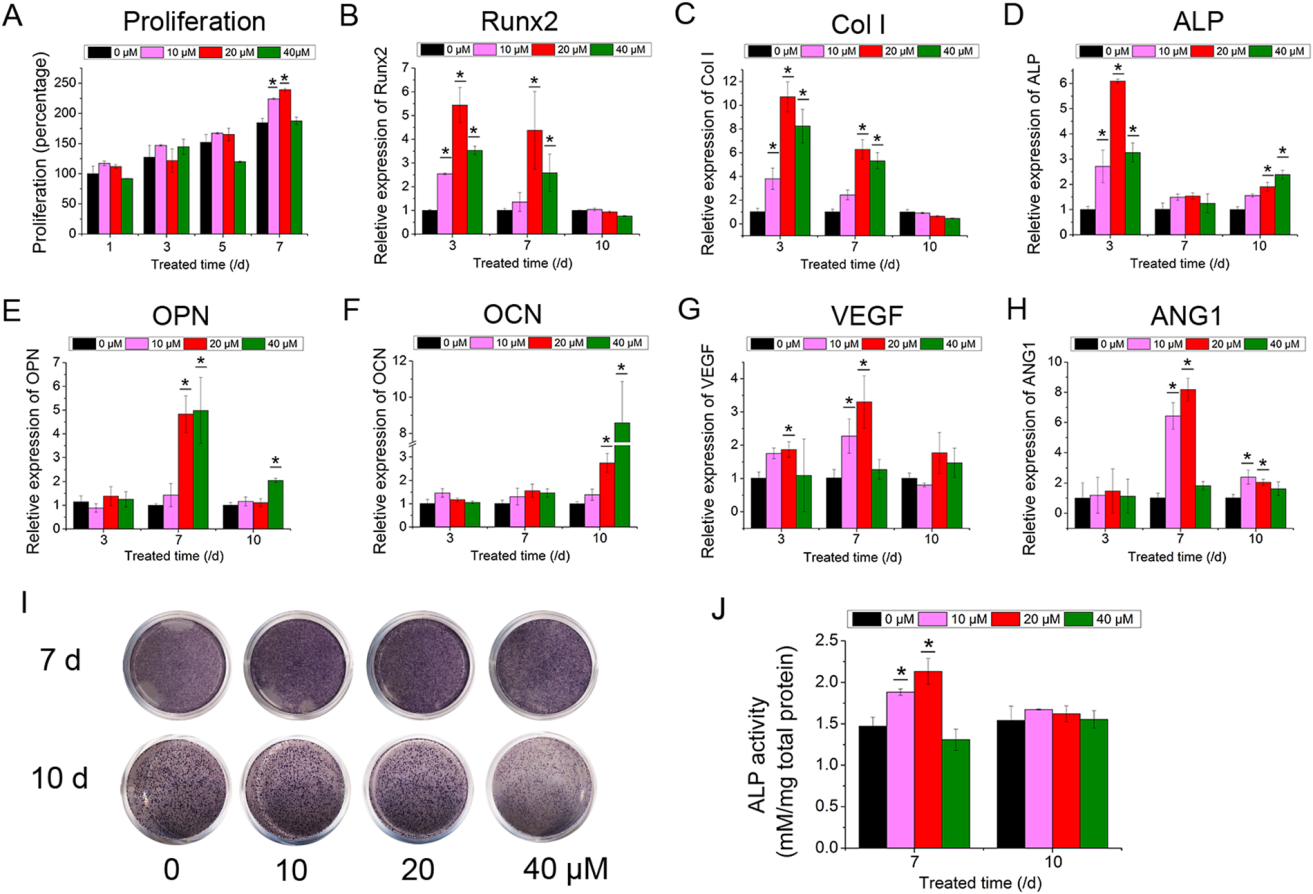
The proliferation, osteoblastic differentiation and angiogenic factor expression effect of icariin on BMSCs. (**A**) Proliferation of BMSCs treated with icariin at concentrations of 10, 20 and 40 µM. (**B**–**F**) Real-time PCR analysis of Runx2, Col I, ALP, OPN, OCN, VEGF and ANG1 mRNA in BMSCs treated with icariin at concentrations of 10, 20 and 40 µM. (**I**) ALP staining of BMSCs after treatment with icariin on days 7 and 10. (**J**) Quantitative ALP activity of BMSCs treated with icariin measured by the pNPP assay on days 7 and 10. (*p < 0.05, n = 3).

The MTT assay showed there was no significant positive effect on the cell proliferation of BMSCs in the icariin-treated groups, except on day 7 (Fig. 1A). The mRNA expression of runt-related transcription factor 2 (Runx2), collagen I (Col I), ALP, osteopontin (OPN) and osteocalcin (OCN) in BMSCs was detected and was found to be improved by icariin at concentrations of 10, 20 and 40 µM, especially in the case of the 20 μM group (p < 0.05, Fig. 1B,C,D,E,F). The expression of Runx2 and Col I mRNA was up-regulated by icariin at 20 and 40 μM on day 3 and 7 (p < 0.05, Fig. 1B,C). Although the expression of ALP mRNA fluctuated from dayd 3 to 10, it was induced on days 3 and 10 (p < 0.05, Fig. 1D). As an intermediate marker for osteogenic differentiation, OPN was induced from day 7 to day 10 by icariin, and its expression peaked at day 7 (p < 0.05, Fig. 1E). OCN, a later marker for osteogenic differentiation, was finally induced on day 10 (p < 0.05, Fig. 1F). Both the quantitative ALP activity and ALP staining assays showed that although ALP activity was not significantly induced on day 10, it was increased on day 7 in the 20 μM group (Fig. 1I,J). Similar to the expression of osteogenic genes, the expression of vascular endothelial growth factor (VEGF) and angiopoietin 1 (ANG1) mRNA was promoted by icariin, especially at 20 μM. The expression of ANG1 mRNA was induced until day 7 after treatment with icariin (p < 0.05, Fig. 1G,H).

These results suggest that the osteoinduction of icariin occurs in a concentration-dependent manner at an optimal concentration of 20 μM.

### Effect of icariin on the expression of osteoclastic genes and the formation of osteoclasts

**Figure 2:**
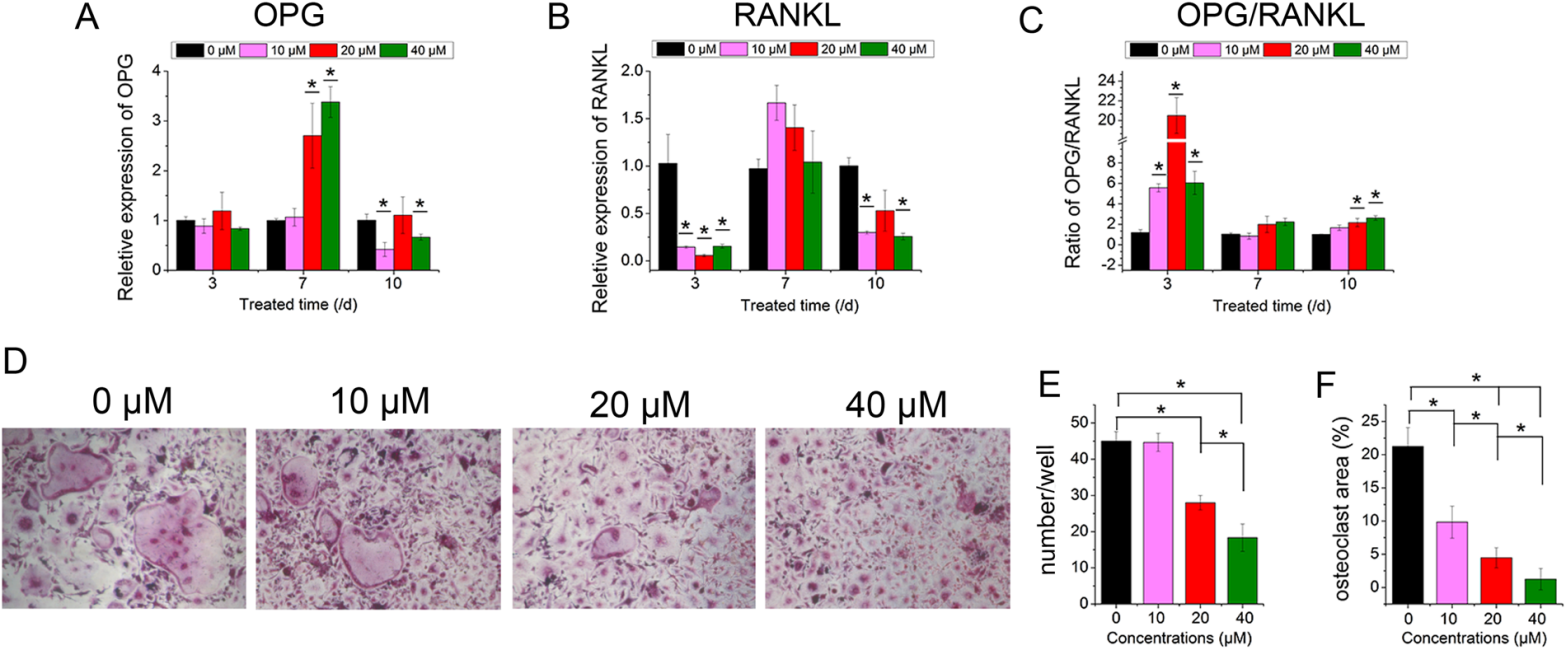
Effect of icariin on the expression of osteoclastic genes and formation of osteoclasts. (**A**,**B**) Real-time PCR analysis of OPG and RANKL mRNA in BMSCs treated with icariin at concentrations of 10, 20 and 40 µM. (**C**) The ratio of OPG/RANKL. (**D**) Formation of osteoclasts. (**E**) Number of osteoclasts. (**F**) Area of osteoclasts. (*p < 0.05, n = 3).

Real-time PCR showed that although icariin did not induce the expression of OPG mRNA on day 3, it promoted the ratio of OPG/RANKL on day 3 by inhibiting the expression of RANKL (Fig. 2A,B,C). Subsequently, the ratio of OPG/RANKL fluctuated to the normal level of the 0 µM group on day 7 and showed a slight increase in the 20 µM and 40 µM groups on day 10, also by inhibiting the expression of RANKL mRNA (Fig. 2B,C). The area and number of osteoclasts decreased in the icariin-treated groups. Moreover, with the increased concentration of icariin, fewer osteoclasts formed, indicating that it inhibits the formation of osteoclasts.

### The release of icariin from the CPC scaffolds

Since the optimal concentration for osteoinduction of icariin was 20 μM *in vitro*, the concentration of icariin applied in the CPC scaffolds at the multiple of 10, 100, and 1 000 folds of that *in vitro* were 200, 2 000, 20 000 μM respectively. The compounds of the CPC loaded with icariin (ICA for short) at concentrations of 0, 20, 200 or 2000 μM are described as CPC, CPC/ICA-20, CPC/ICA-200, CPC/ICA-2000 for short.

**Figure 3:**
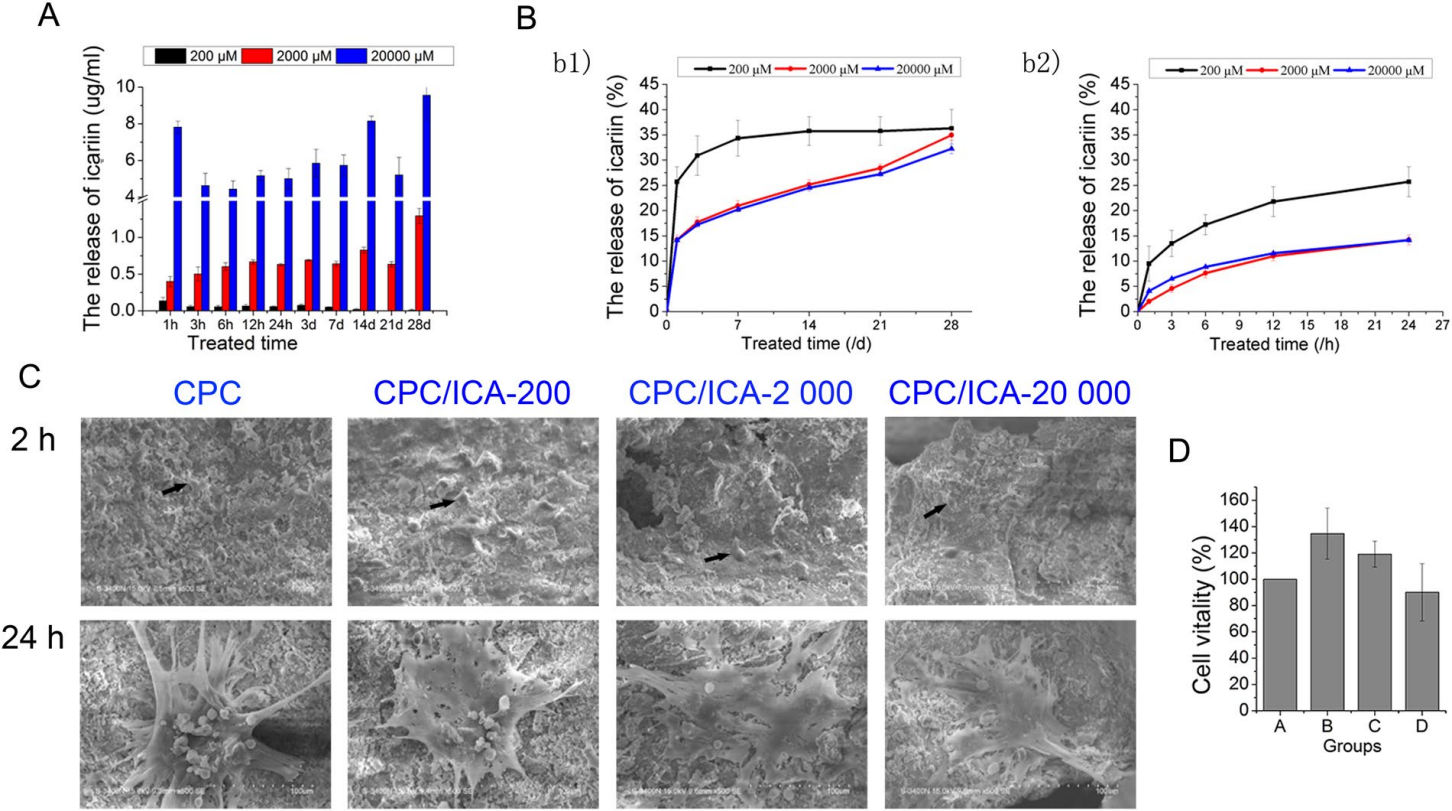
Release of icariin loaded in CPC at concentrations of 200, 2 000 and 20 000 μM, and the morphology, adhesion, and proliferation of BMSCs seeded on CPC with or without icariin. (**A**) Absolute release amount. (**B**) Cumulative release of icariin in percent. (**C**) SEM analysis of BMSCs seeded on samples CPC, CPC/ICA-200, CPC/ICA-2 000, CPC/ICA-20 000 for 2 h and 24 h. Scale bar = 100 μm. (**D**) The vitality of BMSCs on samples CPC, CPC/ICA-200, CPC/ICA-2 000 and CPC/ICA-20 000.

The release of icariin from CPC scaffolds at different concentrations is presented in terms of absolute release amount (Fig. 3A) and cumulative release percentage of three different concentrations (Fig. 3B) when the scaffolds were incubated *in vitro*. In terms of release dose, the release of icariin gradually decreased in CPC/ICA-200 group, and the release dose of icariin fluctuated between 0.5 ug/ml and 1.3 ug/ml in CPC/ICA-2 000 group, while it fluctuated between 4 ug/ml and 10 ug/ml in CPC/ICA-20 000 group (Fig. 3A). While in the cumulative release percentage, it was demonstrated a one-phase exponential release at the first day, the release rate of icariin in the CPC/ICA-200 group reaching to 25%, and it reaching to nearly 15% in both CPC/ICA-2 000 and CPC/ICA-20 000 groups. Though the release of icariin reached to a plateau stage at day 7 in CPC/ICA-200 group, icariin in both CPC/ICA-2 000 and CPC/ICA-20 000 groups demonstrated slow, but linear release during 4 weeks from the second day. To be specific, we also explore the release efficiency of icariin in 24 h. It was showed that there were all fast release rates at the initial 1 h in three groups. The release rate reaching to 10% in CPC/ICA-200 group, then it was gradually reduced in the following hours. Though the absolute release amount of icariin in both CPC/ICA-2 000 and CPC/ICA-20 000 groups are higher than that of CPC/ICA-200 group, these three groups have a similar trend in release efficiency in 24 h. These results indicated that CPC scaffold can be considered as sustained slow release vehicles and that the release rate can be tailored according to the desired to a limited extent.

### Morphology, adhesion and proliferation of seeded BMSCs

It is generally known that cell adhesion and spreading on material surfaces play a crucial role in cell proliferation and differentiation, as well as in osteogenesis *in vivo* ^20^.

Scanning electron microscopy (SEM) was applied to observe the morphology of BMSCs cultured on CPC scaffolds loaded with or without icariin for 2 and 24 h. Though the BMSCs attached on CPC, CPC/ICA-200, CPC/ICA-2 000, and CPC/ICA-20 000 composite materials at 2 h showed little difference in shape, the BMSCs appeared flat and spread out, similar to a polygon at 24 h in four groups, whereas BMSCs on CPC scaffolds had fewer branches with the increasing of icariin concentration (Fig. 3C).

The proliferation of BMSCs cultured on CPC scaffolds with or without icariin was measured by a CCK-8 assay on day 3 (Fig. 3D) and expressed in the form of cell viability in percentage. It was showed that there were no significant differences in cell viability in the CPC/ICA-200 and CPC/ICA-2 000 groups compared with the CPC group.

### Histological analysis of CPC/ICA in nude mice

**Figure 4:**
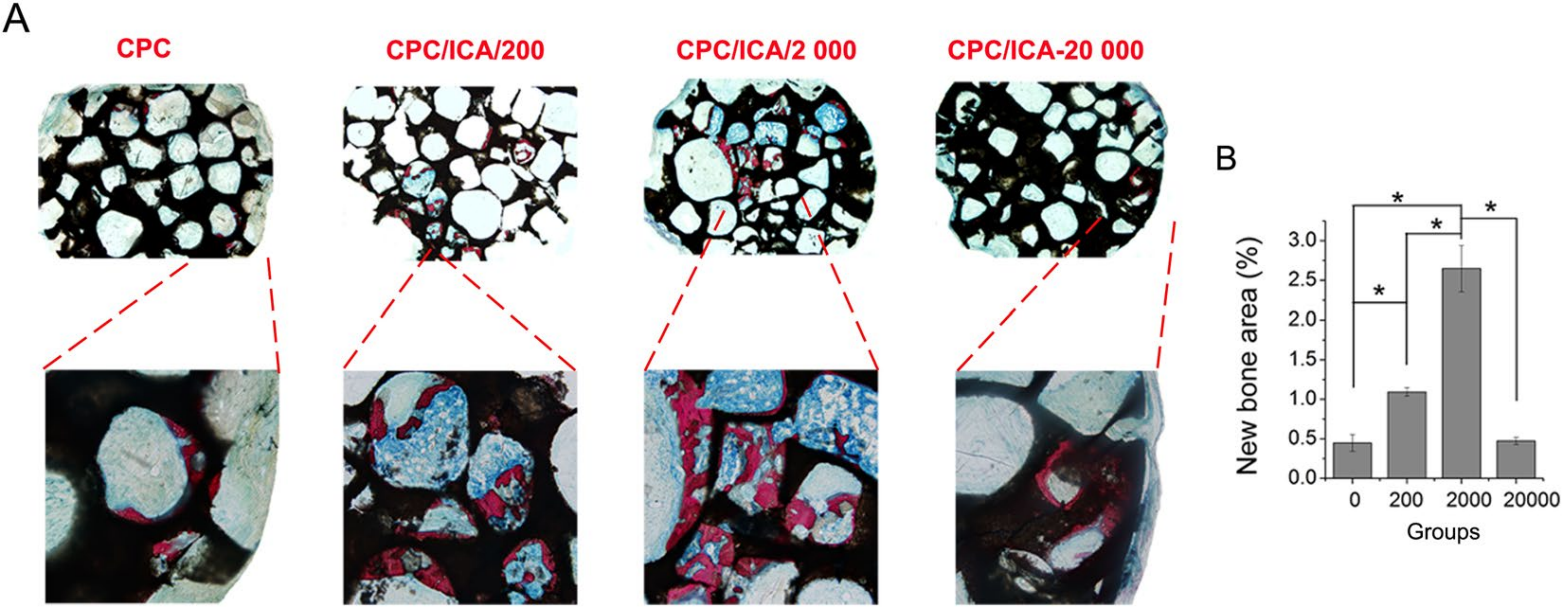
Osteogenic effect of local icariin on BMSCs in CPC scaffolds implanted in nude mice. On these nondecalcified sections, red areas represent newly formed bone, blue areas belong to collagen fibers, and black areas represent undegraded CPC scaffolds. (**A**) Histological images of bone formation for group CPC, CPC/ICA-200, CPC/ICA-2 000, and CPC/ICA-20 000 (Line 1: ×40; Line 2: ×100); (**B**) The percentage of new bone area was assessed by histomorphometric analysis (*p < 0.05).

Subsequently, the CPC composite materials loaded with BMSCs were implanted into nude mice to explore their effect on ectopic bone formation. A histological examination was performed using ectopic implants extracted from the backs of nude mice 8 weeks after implantation. A histological analysis of the CPC compounds using van Gieson’s picro fuchsine stain showed that all of the implants were encapsulated in a fibrous capsule and were colonized with trabecular bone within the cement in the intergranular spaces (Fig. 4A). The percentage of the area of newly formed trabecular area was 2.65 ± 0.29% in the CPC/ICA-2 000 groups, which was higher than that in the CPC (0.44 ± 0.11%), CPC/ICA-200 (1.09 ± 0.05%), and CPC/ICA-20 000 (0.47 ± 0.04%) groups.

These results suggest that the osteoinduction of icariin loaded in CPC scaffolds occur in a concentration-dependent manner with an optimal concentration of 2 000 μM.

### Fluorochrome-labeling histomorphometric analysis

Since the newly formed bone was greatest in the CPC/ICA-2 000 group compared to the other groups in nude mice, the CPC/ICA-2 000 compound was selected to repair calvarial defects in OVX rats.

**Figure 5:**
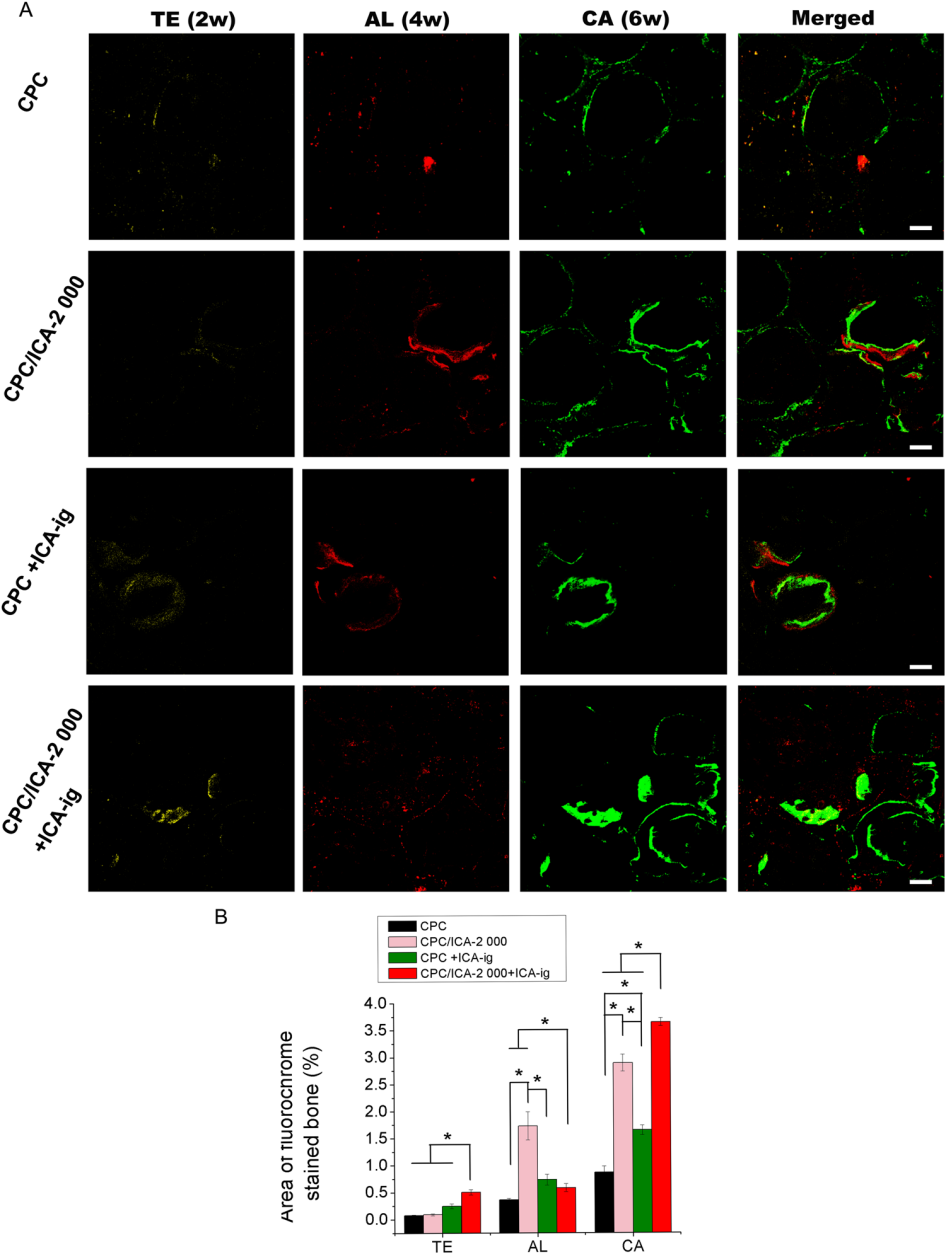
Sequential fluorescent labeling of TE, AL and CA for CPC, CPC/ICA-2 000, CPC +ICA-ig, and CPC/ICA-2 000 +ICA-ig groups. The images in yellow (TE), red (AL) and green (CA) indicated the rate of bone formation and mineralization at 2, 4 and 6 weeks after the operation, respectively. Merged images of the three fluorochromes for the same group. Scale bar = 100 μm. (**B**) The percentage of TE, AL and CA staining by histomorphometric analysis (*p < 0.05).

In the present study, to observe the generation of new bone in calvarial defects in OVX rats, a fluorescent labeling analysis was performed on the samples for 8 weeks. As shown in Fig. 5, the different fluorescent labels representing new bone formation and mineralization were observed at 2, 4, and 6 weeks after the operation. After 2 weeks, the percentage of TE labeling (yellow) in the CPC/ICA-2 000 +ICA-ig group (0.50 ± 0.05%) was higher than that in the CPC +ICA-ig group (0.24 ± 0.05%) (p < 0.05), whereas for the CPC and CPC/ICA-2 000 groups, TE labeling was hard to detect at this early stage. After 4 weeks, a higher percentage of AL labeling (red) was observed in the CPC/ICA-2 000 (1.73 ± 0.26%) and CPC +ICA-ig groups (0.73 ± 0.10%) compared to the CPC (0.36 ± 0.03%) and CPC/ICA-2 000 +ICA-ig groups (0.58 ± 0.07%) (p < 0.05), and there was also a significant difference between the CPC and CPC/ICA-2 000 +ICA-ig groups (p < 0.05). After 6 weeks, the percentage of CA labeling (green) in the CPC/ICA-2 000 (2.89 ± 0.16%) and CPC/ICA-2 000 +ICA-ig groups (3.65 ± 0.08%) was higher than that in the CPC (0.87 ± 0.12%) and CPC +ICA-ig groups (1.65 ± 0.09%), and there was also significant difference between the CPC and CPC +ICA-ig groups (p < 0.05).

### Histological analysis of bone regeneration

**Figure 6:**
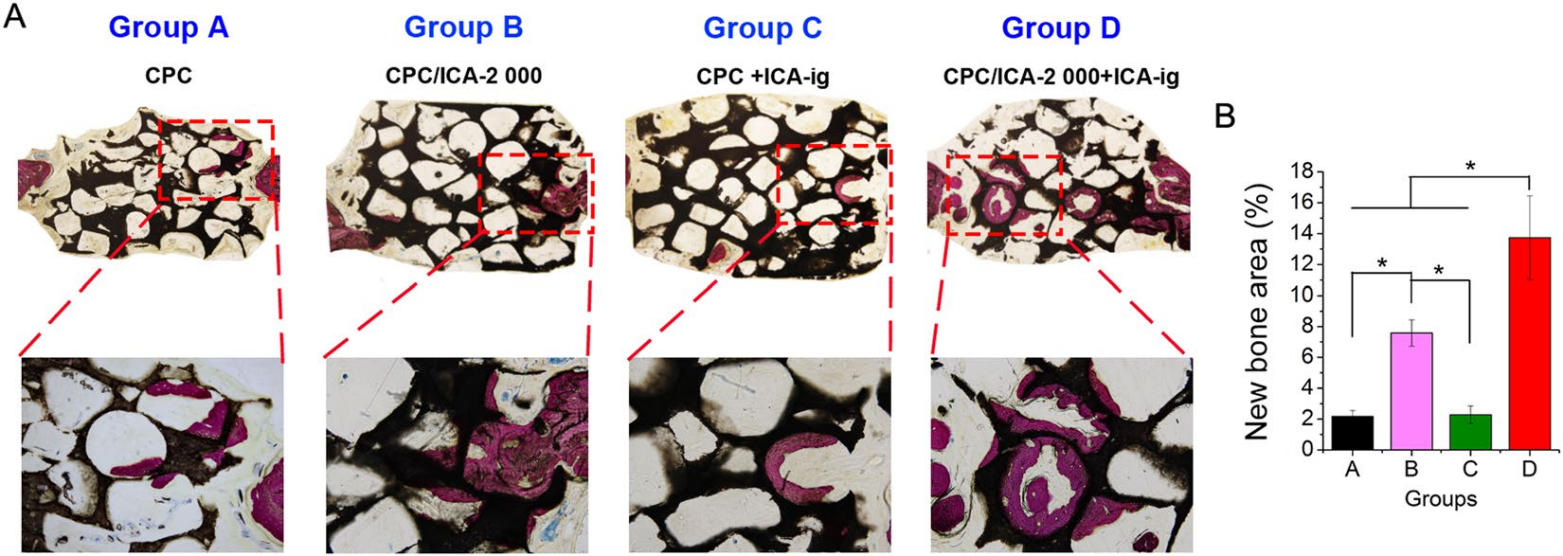
Histological images of newly formed bone in calvarial defects for groups CPC (Group A), CPC/ICA-2 000 (Group B), CPC +ICA-ig (Group C) and CPC/ICA-2 000 +ICA-ig (Group D) that were taken 8 weeks after the operation (Line 1: ×40; Line 2: ×100). On these nondecalcified sections, red areas represent newly formed bone, and black areas belong to undegraded CPC scaffolds. (**B**) The percentages of new bone area were assessed by histomorphometric analysis (*p < 0.05).

The undecalcified specimens stained with van Gieson’s picro fuchsine showed that there was little newly formed bone, and the bone commencing only from the periphery of the host bone, in the CPC (Group A, 2.18 ± 0.38%) and CPC +ICA-ig groups (Group C, 2.28 ± 0.56%) 8 weeks after the operation; By contrast, much more newly formed bone was detected in the CPC/ICA-2 000 (Group B, 7.59 ± 0.86%) and CPC/ICA-2 000 +ICA-ig groups (Group D, 13.73 ± 2.71%), especially in the CPC/ICA-2 000 +ICA-ig group, with new bone formation observed in the center and periphery of the bone defect. Moreover, the area of newly formed bone in the CPC/ICA-2 000 +ICA-ig group was much greater than that in the other groups (p < 0.05) (Fig. 6).

### Microfil perfusion analysis of angiogenesis

**Figure 7:**
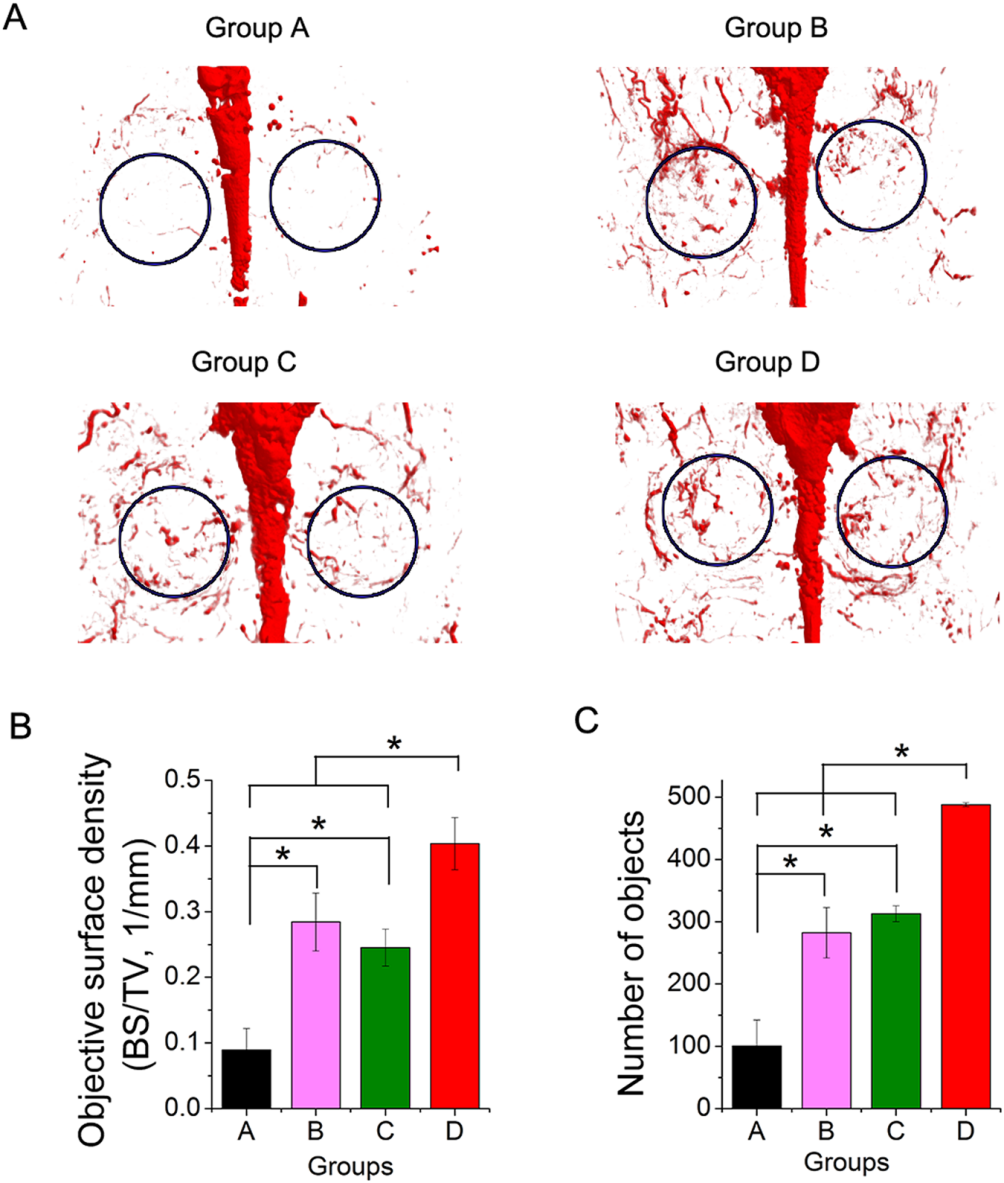
Histological images of newly formed blood vessels in calvarial defects for groups CPC(Group A), CPC/ICA-2 000 (Group B), CPC +ICA-ig (Group C) and CPC/ICA-2 000 +ICA-ig (Group D) that were taken at 8 weeks after the operation. (**B**,**C**) The objective surface density and objects number of newly formed blood vessel were assessed by histomorphometric analysis (*p < 0.05).

To outline blood vessels and evaluate blood vessel formation, 8 weeks after the surgery, the rats were perfused with Microfil (Flowtech, USA) after euthanasia. The area of newly formed blood vessels is represented as the objective surface density and the number of objects in the calvarium defect. The objective surface density in the CPC/ICA-2 000 (Group B, 0.28 ± 0.04%), CPC +ICA-ig (Group C, 0.25 ± 0.03) and CPC/ICA-2 000 +ICA-ig groups (Group D, 0.40 ± 0.04%) was significantly larger than in the CPC group (Group A, 0.09 ± 0.03%) (Fig. 7A) (p < 0.05), while the largest objective surface density was detected in the CPC/ICA-2 000 +ICA-ig group (Fig. 7B, p < 0.05). A similar trend was observed for the object number of the newly formed blood vessels. These results demonstrate that the extent of blood vessel growth in the CPC/ICA-2 000, CPC +ICA-ig and CPC/ICA-2 000 +ICA-ig groups was markedly greater than that in the CPC group. Furthermore, the CPC/ICA-2 000 +ICA-ig group exhibited the most significant improvement, and there were also significant differences between the CPC/ICA-2 000, CPC +ICA-ig and CPC groups (Fig. 7A,B,C).

### Parameters of the OVX rats

**Figure 8:**
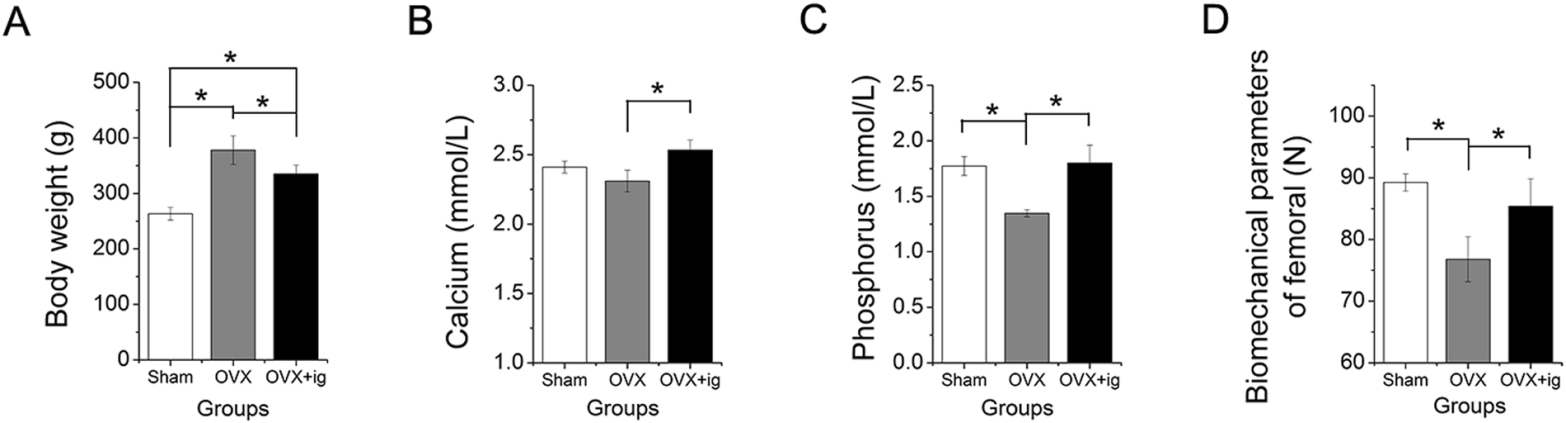
The induced effect of icariin on the weight (**A**), serum calcium (**B**), and serum phosphorus (**C**) content, and femoral biomechanical parameters (**D**) in OVX rats (*p < 0.05).

The body weight of the OVX rats (378 ± 25.88 g) was significantly higher than that of the sham-operated control (sham) rats (263.33 ± 11.55 g). After 12 weeks of a gavage of icariin, the body weight of the OVX rats decreased to 335 ± 16.04 g, which was significantly lower than that of the OVX rats without the gavage (Fig. 8A, *p < 0.05).

The serum assay indicated a reduction in serum calcium (Ca) and phosphorus (P) levels in the OVX rats when compared to the sham group. Statistically significant increases in the levels of serum Ca and P were observed in the OVX rats gavaged with icariin (OVX + ig), which nearly reached the levels of sham group (Fig. 8B, C, p < 0.05), indicating that icariin induced a recovery of the reduced levels of serum Ca and P in the OVX rats.

Similar to the results of the serum assay, the biomechanical strength of the femur in the OVX + ig group increased significantly compared to the OVX group and was comparable to the sham group (Fig. 8D, p < 0.05).

## Discussion

In osteoporosis patients, the balance of bone formation and desorption is disrupted due to the reduced proliferation and osteoblastic differentiation of BMSCs^21^, resulting in a limited capacity for bone regeneration. Epimedium-derived flavonoids, such as icariin, genistein, and daidzein, are proven to stimulate bone formation^18, 22, 23^. Since icariin is more potent than genistein in promoting osteoblast differentiation and mineralization *in vitro* ^24^, it was selected for the observation of its effect on bone repair in osteoporosis in this study.

Icariin, the main active ingredient extracted from *Herba Epimedii*, has a variety of pharmacological properties, including anti-tumor effects, immune system enhancement, improving cardiovascular function and endocrine regulation^25–28^. Importantly, it has been demonstrated that icariin stimulated the osteogenic differentiation of stem cells^16, 29–32^ and promoted osteogenesis *in vivo* ^18, 33^. Furthermore, icariin treatment increased bone density, maximum load and bending strength of femora, enhanced its resistance to external shocks and suppressed the loss of bone mass in OVX rats^14^, which indicates that systemic administration of icariin might play a positive role in bone defect regeneration and bone development. To verify this effect, the osteogenesis and angiogenesis effects of icariin for osteoporosis *in vitro* and *in vivo* was investigated.

In this study, BMSCs derived from OVX rats were treated in advance with icariin at a series of concentrations to determine an appropriate concentration for *in vitro* osteogenesis by detecting cell proliferation, osteogenesis activity and angiogenic factor expression in BMSCs. Our previous study demonstrated that icariin improved the osteogenic differentiation of BMSCs derived from young rats^15^. According to this result, icariin at concentrations of 10, 20 and 40 μM was selected for observation and showed a significant effect on the proliferation of BMSCs derived from OVX rats at day 7. Meanwhile, icariin significantly induced the expression of osteogenic and angiogenic genes, especially at 20 μM. These genes included ALP, the expression of which was verified by ALP staining and quantitative activity analysis. Thus, the optimal concentration of icariin *in vitro* for the BMSCs cultured in the present study was 20 μM, which was in accordance with the concentration applied *in vivo*.

In osteoporosis, the enhancement of osteoclasts and increase in active bone resorption lead to microarchitectural deterioration and bone structure collapse, bringing about the loss of bone mass^34, 35^. Osteoclasts are differentiated under the control of cytokines, such as macrophage colony-stimulating factor (M-CSF), receptor activator of NF-кB ligand (RANKL), and osteoprotegerin (OPG). RANKL is known as a key cytokine that binds to its receptor RANK in the hematopoietic lineage and then stimulates the activity and recruitment of osteoclasts^36^. OPG is recognized as a competitive receptor of RANKL that might prevent the activation of RANK, resulting in decreased osteoclast activity^37^. The association between osteoclasts and osteoblasts in bone metabolism is greatly affected by the balance between RANKL and OPG, which modulates the level of bone resorption^38^. Therefore, the ratio of OPG/RANKL in bone microenvironment is crucial for the activities of osteoclasts, and upregulating the ratio of OPG/RANKL will decrease the differentiation and maturation of osteoclasts^39^. In the present study, icariin promoted the gene expression ratio of OPG/RANKL of BMSCs by up-regulating OPG expression and inhibiting RANKL expression (Fig. 2A,B). Though there was a fluctuation of OPG/RANKL ratio during icariin treatment, it was still greater in both 20 and 40 μM groups than the control group at day 10, which indicated the progress of osteogenesis. Furthermore, the formation of osteoclasts was inhibited by icariin, and less formation of osteoclasts was found when increasing the icariin concentration from 10 to 40 μM (Fig. 2D,E,F). Overall, the action of icariin in improving osteogenic differentiation and angiogenic gene expression in BMSCs and inhibiting the formation of osteoclasts makes it an excellent candidate for bone regeneration in osteoporosis patients.

When used as an artificial extracellular matrix, biomaterial scaffolds act as basic frameworks providing support and living space for cellular adhesion, growth, division, and metabolism and forming a new structure for the purpose of tissue engineering. Furthermore, they are also used as carriers for osteogenic factors or drugs. In the perspective of bionics, biomaterial scaffolds used for bone tissue regeneration should possess a porous structure to mimic natural bone tissues, such as cancellous bone, a grid architecture formed by trabecular bone and Folkman’s tube that is interconnected to exchange nutrients. A degree of porosity 75% and pore size of 90~300 μM are generally believed to be preferable for cell growth, helping to facilitate the formation of new bone without simultaneously affecting its mechanical strength. Ceramic materials, such as CPC^40^, hydroxyapatite (HAp)^41^, and tricalcium phosphate (TCP)^42^, have been extensively utilized in clinical application due to their intrinsic superiority. The CPC scaffolds employed in this experiment were a paste mixture of calcium phosphate and other calcium-containing compound powders with a suitable proportion of water or aqueous solution that was concreted by exposure to a high or physiological temperature to form 3D scaffolds with a certain structural strength. A previous study demonstrated that albumin protein penetrates well into the porous crystal network of the CPC, being adsorbed onto the porous crystal network of the CPC, being adsorbed onto the surface of CPC crystals. This is highly dependent on the liquid-to-powder (L/P) ratio used for the CPC preparation as well as on the initial particle size used for the CPC powder, because both factors significantly influence the micro/nanostructure and the pore size distribution of the CPCs. The higher the L/P ratio used, the greater is the penetration of the protein. Furthermore, it was observed that when initial particle sizes were larger the pores inside the CPCs^43^. So far, there have been no reports of release efficiency of small molecule drugs loaded on CPC. In this study, the release of icariin at different concentrations in CPC scaffolds showed a one-phase exponential release at the first day. Though the release of icariin reached to a plateau stage at day 7 in CPC/ICA-200 group, icariin in both CPC/ICA-2 000 and CPC/ICA-20 000 groups demonstrated slow, but linear release during 4 weeks from the second day (Fig. 3b1). In specific, it was showed that there were all fast release rates at the initial 1 h in three groups, while the release rate was gradually reduced in the following hours. Though the absolute release amount of icariin in both CPC/ICA-2 000 and CPC/ICA-20 000 groups are higher than that of CPC/ICA-200 group, these three groups have a similar trend in release efficiency in 24 h (Fig. 3b2). These results indicated that CPC scaffold can be considered as sustained slow release vehicles.

To repair bone defects in rats with osteoporosis effectively, it is very important to select an appropriate drug administration. According to previous studies, when administered systemically, icariin effectively promotes bone fracture healing in OVX rats by gathering physiological factors, enhancing calcium absorption, and promoting osteogenic differentiation, osteoblastic cell maturity and fracture revascularization in order to accelerate the formation of a callus^18^. In the present study, a system of local delivery and systemic administration was selected to observe the repair effect of icariin. Icariin was systematically administered daily by gavage at a dose of 25 mg/kg body weight^14^. The results showed that local delivery of icariin combined with systemic administration (Group D) achieved the best bone repair effect, followed by local delivery of icariin (Group B) (Fig. 6). Although systemic administration of icariin with (Group C) did not significantly promote new bone formation, it promoted angiogenesis of the calvarial defect (Fig. 7C).

Bone regeneration is sustained by angiogenesis. Blood vessels generate a metabolic microenvironment, maintaining perivascular osteoprogenitors and mediating the growth of the vasculature, which is coupled with osteogenesis^44^. In the present study, the expression of angiogenic factors such as VEGF and ANG1, in BMSCs was promoted by icariin. VEGF plays an important role in the proliferation and differentiation of endothelial progenitor cells (EPC). Moreover, the exogenous administration of VEGF promotes angiogenesis and accelerates bone healing^45^. ANG1, an endothelial growth factor, functions as a ligand for the endothelial-specific receptor tyrosine kinase Tie2^46^. The Tie2 and VEGF receptor (VEGFR) pathways seem to work in a complementary and coordinated fashion during vascular development, with VEGF acting at the early stages of vessel development^47, 48^, and ANG1 acting later to promote angiogenic remodeling^49^. In present study, icariin delivered locally (Group B) or administered systemically (Group C), promoted angiogenesis in the bone defect area, while the local sustained release of icariin combined with systemic administration (Group D) achieved the best effect. Hence, the system combining local delivery and systemic contributes to new bone formation and angiogenesis. Thus, it could be speculated that the angiogenesis induced by icariin might assist bone repair.

To further explore the mechanism of bone formation occurring in the icariin systemic administration and local delivery groups, we examined the weight, serum calcium and phosphorus level, and femur mechanical strength of the OVX rats. The results showed that icariin effectively suppresses the increase in the body weight of OVX rats, improves serum calcium and phosphorus levels and increases the bending strength of the femur (Fig. 8), suggesting that it slows down the progress of osteoporosis in OVX rats, which further contributes to new bone formation in the calvarial defect. Moreover, the endometrium is an exquisitely hormone-sensitive tissue and a principal target of estrogen and progesterone. According to a previous study, icariin treatment in OVX rats could increase uterus thickness, endometrial thickness, endometrial glands, and vaginal epithelium cell layers and thickness, which indicated that icariin possessed estrogen-like effects. However, icariin was demonstrated to be lack of uterotrophic activity, which could be beneficial for reducing the risk of endometrial, breast, and ovarian cancer associated with estrogen treatment^50^.

**Figure 9:**
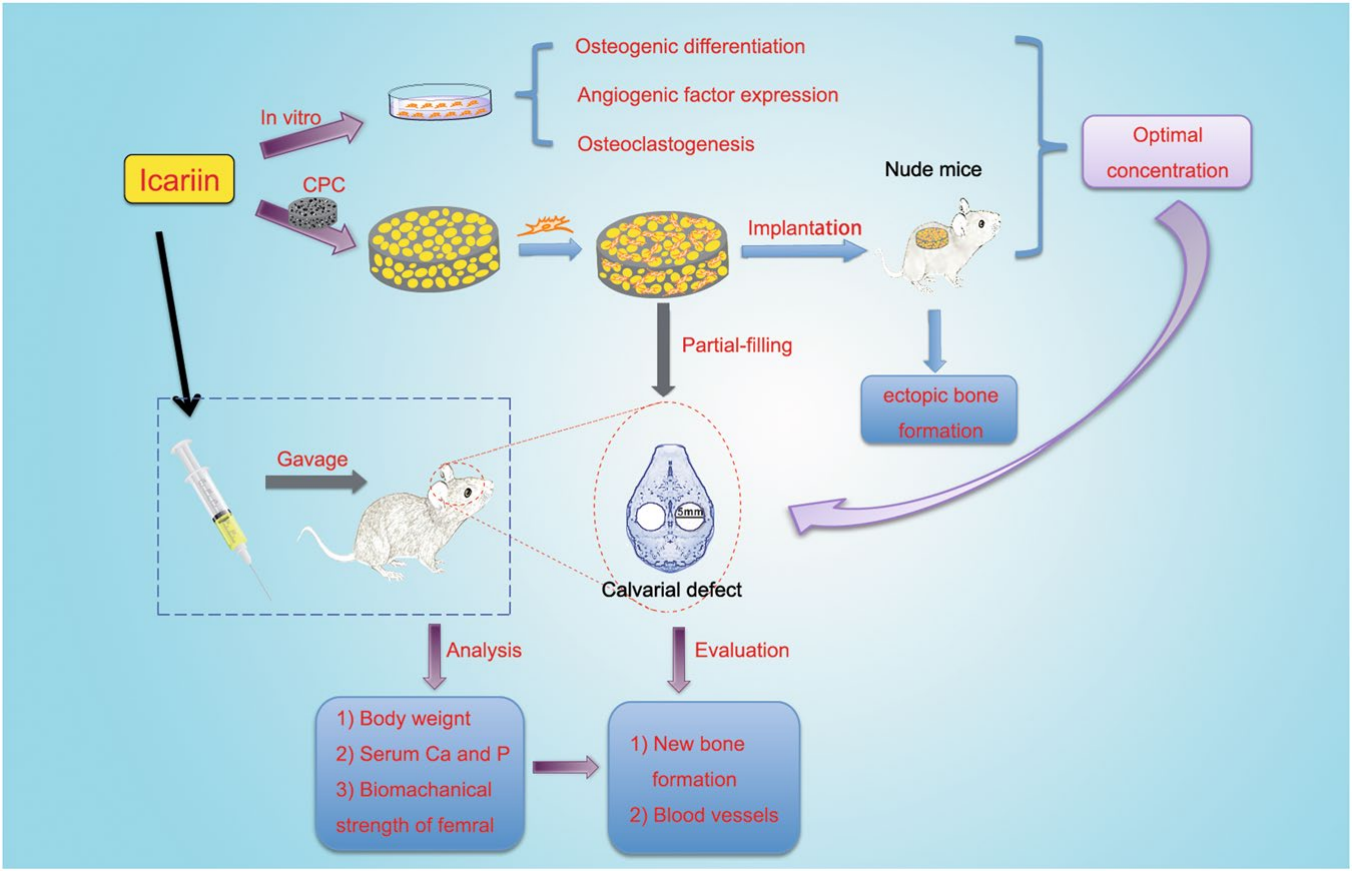
Schematic diagram of depicting the conceptual framework.

Above all, we conclude that icariin promotes the repair of bone defects in osteoporosis by promoting the proliferation, osteogenesis and angiogenic factor expression of BMSCs and inhibiting osteoclast differentiation, while angiogenesis indirectly promotes osteogenesis (Fig. 9).

## Conclusion

In the present study, we demonstrate that icariin promotes the osteogenic differentiation and expression of angiogenic factors in BMSCs. In addition, it inhibits osteoclastic differentiation. In the OVX calvarial defect model, icariin loaded on CPC scaffolds enhances both osteogenesis and angiogenesis, while the system of local sustained release of icariin combined with systemic administration achieves a better effect for bone defect regeneration. The present study may provide a promising new strategy for repairing bone defects and suggests that icariin is a candidate drug that it achieves better repair with systemic administration.

## Materials and Methods

### Animals and Ovariectomy

This study was conducted in accordance with the regional Ethics Committee guidelines, and the experimental protocols were approved by the Animal Experimental Ethical Committee of Shanghai Ninth People’s Hospital affiliated to Shanghai JiaoTong University. Thirty-six female Sprague-Dawley (SD) rats aged 3 months and weighing 220 ± 20 g were obtained from Shanghai SLAC Experimental Animal Center (Shanghai, China). Thirty animals underwent surgical ovariectomy under anesthesia using chloral hydrate (Shenggong, Shanghai, China; 0.04 mL/kg body weight, intraperitoneally), while six rats were sham-operated (Sham group). The rats were then housed in a temperature-controlled room (21 °C) with a relative humidity of 60% under a 12/12-h light/dark cycle.

### Cell culture

Twelve weeks after the ovariectomy, the rats in both groups were sacrificed. The humerus was isolated from the OVX rats. The bone marrow was flushed out with Dulbecco’s modified Eagle’s medium (DMEM, HyClone, Logan, USA) supplemented with 100 units/mL penicillin and 100 μg/mL streptomycin (HyClone, Logan, USA). The whole washouts were collected and centrifuged (1800 *g*, 10 min). Next, the pellet was mixed with DMEM and then plated in a culture flask and maintained at 37 °C under 5% atmosphere. The primary rat BMSCs were trypsinized with 10% trypsin-EDTA (HyClone, Logan, USA) and passaged. BMSCs from passage 2 to 3 were used for our experiments.

### Cell proliferation assay

For the cell proliferation assay, BMSCs were seeded onto 96-well plates at a density of 5 × 10^3^ cells/well. After 24 h of incubation, the BMSCs were treated with icariin at concentrations of 10, 20 and 40 μM for 1, 3, 5 and 7 days. Rats in the control group (0 μM) were treated with an equal volume of vehicle (1 μL DMSO/ml medium). Cell proliferation was assessed by an MTT or Cell Counting Kit-8 (CCK8) (Dojin, Shanghai, China) assa according to the manufacturer’s instructions y. The absorbance was determined at 590 nm or 450 nm with an ELX Ultra Microplate Reader (BioTek, Burleigh, QLD, Australia). The percentage of viable cells was obtained by comparing the absorbance of samples with and without icariin. All experiments were performed in triplicate.

### Chemicals

Icariin was purchased from Tauto Biotech Company (Shanghai, China). Reagent kits for the measurement of serum calcium, inorganic phosphorus concentration, and serum alkaline phosphatase activity were obtained from Fortune Bio-medical Engineering Co. Ltd. (Shanghai, China).

### Real-time PCR assay

BMSCs were plated into 12-well plates at a density of 1 × 10^5^ cells/well and incubated for 24 h followed by incubation with icariin at concentrations of 0, 10, 20 and 40 μM. Total RNA was isolated from the cells after 3, 7 and 10 days of icariin treatment using the Trizol reagent (Invitrogen, USA), according to the manufacturer’s recommended protocol. Complimentary DNA (cDNA) was synthesized by means of a cDNA Synthesis Reverse Transcription Kit (Fermentas, Thermo, USA). Real-time PCR assay for runt-related transcription factor 2 (Runx2), collagen I (Col I), osteopontin (OPN), osteocalcin (OCN), vascular endothelial growth factor (VEGF), and angiopoietin-1 (ANG 1) were performed using a Light-Cycler system with SYBR Premix Ex Taq^TM^ (Takara, Japan) according to the manufacturer’s instructions. The conditions for the real-time PCR were as follows: denaturation at 95 °C for 10 s; 50 cycles at 95 °C for 10 s and 60 °C for 30 s; and a final dissociation stage (95 °C for 5 min) added at the end of the amplification procedure. β-Actin was used as the internal control. The data were analyzed using the comparative Ct (2^−ΔΔCt^) method and are expressed as a fold change respective to the control. Each sample was analyzed in triplicate. The primer sequences used in the present study are listed in Table 1.

**Table1:** List of primers used and their respective forward and reverse sequences.

Gene	Forward sequence
	Reverse sequence
β-actin	5′-GTAAAGACCTCTATGCCAACA-3′
	5′-GGACTCATCGTACTCCTGCT-3′
Runx2	5′-ATCCAGCCACCTTCACTTACACC-3′
	5′-GGGACCATTGGGAACTGATAGG-3′
Alkaline phosphatase (ALP)	5′-TATGTCTGGA ACCGCACTGAAC-3′
	5′-CACTAGCAAGAAGAAGCCTTTGG-3′
Collagen type I(COL I)	5′-CTGCCCAGAAGAATATGTATCACC-3′
	5′-GAAGCAAAGTTTCCTCCAAGACC-3′
Osteopontin (OPN)	5′-CCAAGCGTGGAAACACACAGCC-3′
	5′-GGCTTTGGAACTCGCCTGACTG-3′
Osteocalcin(OCN)	5′-GCCCTGACTGCATTCTGCCTCT-3′
	5′-TCACCACCTTACTGCCCTCCTG-3′
Vascular endothelial growth factor (VEGF)	5′-GGCTCTGAAACCATGAACTTTCT-3′
	5′-GCAATAGCTGCGCTGGTAGAC-3′
Angiopoietin-1(ANG1)	5′-GGACAGCAGGCAAACAGAGCAGC-3′
	5′-CCACAGGCATCAAACCACCAACC-3′
Receptor Activator for Nuclear Factor-κ B Ligand (RANKL)	5′-CATCGGGTTCCCATAAAG-3′
	5′-GAAGCAAATGTTGGCGTA-3′
Osteoprotegerin (OPG)	5′-TTGAAATGGCAGTTGATTCCTTT-3′
	5′-TATCCTCTTTCTCAGGGTGCTTG-3′

### Alkaline phosphatase (ALP) activity assay

Quantitative ALP activity and staining assays were performed at day 7 after BMSCs were treated with icariin at concentrations of 0, 10, 20 and 40 μM. Samples from all groups were incubated with p-nitrophenyl phosphate (pNPP) (Beyotime, Suzhou, China) at 37 °C for 30 min. The absorbance values (OD) were recorded at 405 nm to determine ALP activity. Total protein content was assessed using a BCA protein assay kit (Sigma, USA), and the OD values were normalized to bovine serum albumin (BSA, Sigma, USA) standard curve at 590 nm. ALP activity was assessed as the OD value at 405 nm per milligram of total protein. In addition, ALP staining was performed according to the manufacturer’s instructions (Beyotime, Suzhou, China) and was then observed by a digital camera (Eclipse-100, Nikon, Tokyo, Japan). All experiments were performed in triplicate.

### Osteoclast differentiation and TRAP staining

Four-week-old SD rats were used in this study. Primary bone marrow-derived macrophages (BMMs) were isolated from whole bone marrow as described previously^51^. Briefly, the cells were isolated from the femur and tibia bone marrow and cultured for 24 h in a T75 flask in α-MEM supplemented with 10% FBS, 1% penicillin/streptomycin, and 10 ng/mL M-CSF. The non-adherent cells were then removed, and the adherent cells were cultured in an incubator at 37 °C, 5% CO_2_ for another 3–4 days until the cells were fully confluent. The BMM cells were then seeded into a 96-well plate at a density of 8 × 10^3^ cells/well in complete α-MEM supplemented with 30 ng/mL M-CSF, 50 ng/mL RANKL, and different concentrations of icariin (0, 10, 20, and 40 μM). The cell culture medium was replaced every 2 days until mature osteoclasts formed after 5 days of culture. The cells were stained for TRAP using a Diagnostic Acid Phosphatase kit. TRAP-positive cells with more than three nuclei were counted under a microscope. We quantified the total area of TRAP-positive regions and the total number of osteoclasts on five randomly selected fields of view for each sample.

### Preparation of the implantation constructs and the release kinetics of icariin from CPC scaffolds

According to growth factor studies, the concentration of BMP2 applied in a bone defect model was 10~200-fold greater of that *in vitro* ^52^. In the present study, the concentrations of icariin encapsulated in CPC were selected at multiples of 10, 100 and 1000-folds of the optimized concentration *in vitro* (200, 2 000 and 20 000 μM, respectively).

The CPC scaffold (5-mm diameter and 3 mm long) was loaded with 20 μL of icariin at concentrations of 0, 20, 200 and 2000 μM, respectively, followed by lyophilization to evaporate the DMSO solvent (Sigma, USA). The compounds of the CPC loaded with icariin (ICA for short) at concentrations of 0, 20, 200 or 2000 μM are described as CPC, CPC/ICA-20, CPC/ICA-200, CPC/ICA-2000 for short. One milliliter of SBF was added to each compound and incubated at 37 °C. Then, the supernatant was collected at each selected time point (1, 3, 6, 12, 24 hours and 3, 7, 14, 21, 28 days). Next, the compound was resuspended in fresh SBF and incubated until the next time point. The release of icariin was quantified by an HPLC system (Shimadzu 2010C, USA), and the data are presented in terms of the cumulative release as a function of release time:

$${\rm{Cumulative}}\,{\rm{amount}}\,{\rm{of}}\,{\rm{release}}( \% )=100\times M{\rm{t}}/M\infty $$

Where *M*t is the amount of icariin released from a sample at time t. The total amount of icariin in a sample was calculated and regarded as *M*∞ in this study. Three samples were tested for each group, and the results are reported as average values.

### Morphology, adhesion and vitality of seeded BMSCs

Rat BMSCs were seeded on CPC compound scaffolds at a density of 10 × 10^6^ cells/mL^53^. The samples were collected for SEM assay at 2 h and 24 h after cell seeding, respectively. The samples from each group were fixed in 2.5% glutaraldehyde overnight at 4 °C, washed with PBS three times and then dehydrated by an increasing concentration of ethanol. Finally, these samples were dried by hexamethyldisilazane, sputter-coated with gold and examined by SEM. The proliferation of adherent BMSCs on CPC with or without icariin was detected by the CCK8 assay (as described in 5.3) and expressed in the form of cell viability in percentage. The cell viability was calculated relative to control by using the following formula: (icariin-treated BMSCs group - zeroing OD)/(icariin-untreated BMSCs – zeroing OD).

### Osteogenic effect of icariin on BMSCs in CPC scaffolds in Nude Mice

The nude mice model applied in our study was used to evaluate the effect of icariin loaded on the CPC scaffolds on tissue-engineered bone formation. Rat BMSCs were seeded onto CPC compound scaffolds at a density of 1 × 10^7^ cells/mL. Four dorsal subcutaneous pockets were formed in each anesthetized nude mouse for the insertion of the following four groups of constructs: CPC/BMSCs construct; CPC/ICA-20/BMSCs construct; CPC/ICA-200/BMSCs construct; and CPC/ICA-2000/BMSCs construct. Eight weeks later, the samples were extracted and fixed in 10% buffered formaldehyde. The samples were dehydrated in ascending concentrations of alcohol from 70% to 100%, and then embedded in polymethylmethacrylate (PMMA). Three longitudinal sections of each specimen were prepared as described in our previous study^17^. Then, the samples were stained with van Gieson’s picro fuchsin for histological observation. The area of newly formed bone was quantified from the serial section collected from each sample using a personal computer-based image analysis system (Image-Pro-Plus, Media Cybernetic, USA) and is reported as a percentage of the whole bone defect area.

### Calvarial defect experiments and the administration of icariin

BMSCs were collected and resuspended in DMEM without FBS at a density of 1 × 10^7^ cells/mL, and then 20 μL of the cell suspension was dropped onto the CPC scaffolds and the CPC/ICA-200 compound scaffolds, which were incubated in a 24-well plate at 37 °C for 4 h. Twenty-four OVX rats were allocated into four groups randomly. All rats received a bilateral critical-sized defect model surgery as described in our previous study^1^. Briefly, the animals were anaesthetized by an intraperitoneal injection of pentobarbital (Nembutal 3.5 mg/100 g). Then, a 1.0- to 1.5- sagittal incision was made on the scalp, and the calvarium was exposed by a blunt dissection. Two critical-sized defects were created by using a 5-mm diameter trephine bur (Fine Science Tools, USA). Finally, 48 critical-sized calvarial defects in 12 rats were randomly filled with the following four groups of materials: group A, CPC (A, n = 6); group B, CPC/ICA-200 (B, n = 6); group C, CPC +ICA (ig) (C, n = 6); and group D, CPC/ICA-200 +ICA (ig) (D, n = 6). Rats in groups C and D were treated with icariin (25 mg/kg body weight, daily, i.g.) for 8 weeks. Rats in groups C and D were treated orally with vehicle (water).

### Serum parameters

Serum calcium (Ca), inorganic phosphorus (Pi) concentration, and ALP activity were measured by an automatic analyzer (Ciba-Corning 550, USA) using a diagnostic reagent kit for the determination *in vitro*.

### Measurement of bone mineral density (BMD)

The lumbar spine was cleaned of adhering soft tissues, and the bone mineral density (g/cm2) was measured by dual-energy X-ray absorptiometry (LUNAR Co. Ltd., USA) using the small animal scan mode (Pastoureau *et al*. 1995).

### Biomechanical parameters

The femur was assessed by a loading test of the femoral neck, and a three-point bending test, which were performed using a tensile strength testing machine (Tinius Olsen, PA, USA).

### Sequential fluorescent labeling

Polychrome sequential fluorescent labeling for new bone formation and mineralization was performed over an 8-week observation period in rats according to our previous study [2]. Briefly, the animals were intraperitoneally injected with 25 mg/kg tetracycline hydrochloride (TE, Sigma, USA), 30 mg/kg alizarin red (AL, Sigma, USA), and 20 mg/kg calcium (CA, Sigma, USA), at 2, 4, and 6 weeks after the operation, respectively.

### Histological and histomorphometric observation

The samples of calvarial were dehydrated in ascending concentration of alcohols from 70% to 100%, and then embedded in polymethylmethacrylate (PMMA). Three longitudinal sections for each specimen were prepared as described in our previous study^2^. Firstly, the samples were observed for fluorescent labeling using CLSM (Leica TCS, Germany), and the fluorescent staining for new bone formation and mineralization was quantified. The data with yellow (TE), red (AL), and green (CA) represent the bone regeneration and mineralization at 2, 4, and 6 weeks after operation, respectively. Finally, the samples were stained with von Gieson’s picro fuchsine for histological observation. The area of newly formed bone was quantified from the serial section collected from each sample, using a personal computer-based image analysis system (Image Pro Plus 6.0, Media Cybernetic, USA) and reported as the percentage of the whole bone defect area, respectively.

### Microfil perfusion

To outline blood vessels and evaluate blood vessel formation, 8 weeks after the surgery, the rats were perfused with Microfil (Flowtech, USA) after euthanasia. A long incision was made from the xiphoid down to the abdomen; then, the abdominal aorta was exfoliated and incised, and 10 mL of heparinized saline was perfused. Subsequently, 10 mL of Microfil was perfused at a rate of 2 mL/min following a perfusion with saline^17^.

### *In vitro* observation of the gross specimen blood vessels

To detect blood vessel formation, samples were processed using an alcohol-methyl salicylate clearing sequence. Briefly, samples were decalcified by 10% ethylene diamine tetraacetic acid. Then, digital pictures were acquired (Nikon Digital SLR, D5100, Japan). The areas of the newly formed blood vessels in the bone defect areas were measured using Image J software (National Institute of Mental Health, Bethesda, Maryland, USA).

### Statistical analysis

All experiments were performed a minimum of three times. All measurements are expressed as the mean ± SD. Significant differences between groups were determined using ANOVA (SPSS, v.17.5, USA), while p < 0.05 was considered to indicate statistical significance.
